# Burden of asymptomatic malaria among school-aged children: Protocol for a systematic review and meta-analysis

**DOI:** 10.1371/journal.pone.0359714

**Published:** 2026-09-30

**Authors:** Philmon Dejen, Belay Beyene, Tizazu Zenebe Zelelie, Amanuel Kelem, Abiel Teshome, Mahider Ayalew, Techalew Shimelis

**Affiliations:** 1 Department of Medical Parasitology, School of Medical Laboratory Science, Asrat Woldeyes Health Science Campus, Debre Berhan University, Debre Berhan, Ethiopia; 2 Department of Medical Microbiology, School of Medical Laboratory Science, Asrat Woldeyes Health Science Campus, Debre Berhan University, Debre Berhan, Ethiopia; 3 Department of Clinical Hematology, School of Medical Laboratory Science, Asrat Woldeyes Health Science Campus, Debre Berhan University, Debre Berhan, Ethiopia; 4 Department of Environmental Health, School of Public Health, Asrat Woldeyes Health Science Campus, Debre Berhan University, Debre Berhan, Ethiopia; 5 Department of Biostatistics and Epidemiology, School of Public Health, Asrat Woldeyes Health Science Campus, Debre Berhan University, Debre Berhan, Ethiopia; 6 Department of Medical Laboratory Science, College of Medicine and Health Sciences, Hawassa University, Hawassa, Ethiopia; Bule Hora University, ETHIOPIA

## Abstract

**Background:**

Asymptomatic malaria infection remains a major challenge for malaria control and elimination efforts, particularly in endemic settings such as Ethiopia. School-aged children are increasingly recognized as a potential reservoir for malaria transmission due to frequent exposure to infection and the development of partial immunity that allows infections to remain clinically silent. However, available evidence on the burden, species distribution, and determinants of asymptomatic malaria in this population is fragmented and inconsistent. This systematic review and meta-analysis aim to synthesize evidence on the prevalence, Plasmodium species distribution, and risk factors of asymptomatic malaria among school-aged children in Ethiopia.

**Methods:**

This protocol was developed in accordance with the PRISMA-P guidelines. A comprehensive literature search will be conducted in electronic databases (PubMed, Scopus, CINAHL, and ScienceDirect) and additional sources, including Google Scholar and African Journals Online. Eligible studies will include observational studies reporting asymptomatic malaria among school-aged children in Ethiopia. Two independent reviewers will conduct study selection, data extraction, and quality assessment using the Joanna Briggs Institute critical appraisal tool for prevalence studies. A random-effects meta-analysis will be performed using R software to estimate the pooled prevalence. Heterogeneity will be assessed using Cochran’s Q test and I^2^ statistics, and subgroup analyses will be conducted by diagnostic method, study area, study design, years of actual data collection, and transmission season during data collection. Where sufficiently comparable adjusted estimates are available, associations between prespecified factors and AMI will also be quantitatively synthesized.

**Conclusion:**

The findings will generate comprehensive evidence to support malaria control and elimination strategies in Ethiopia by highlighting the epidemiological importance of school-aged children as a potential reservoir for infection. The results are expected to inform targeted interventions and strengthen school-based malaria surveillance and prevention programs.

## Background

Malaria remains a major public health challenge worldwide, with the greatest burden occurring in sub-Saharan Africa [1]. Ethiopia is among the malaria-endemic countries in the region, where transmission is unstable and heterogeneous owing to variations in altitude, rainfall patterns, ecological conditions, and vector distribution. Both *Plasmodium falciparum* and *Plasmodium vivax* are co-endemic and continue to contribute substantially to malaria morbidity across diverse transmission settings [2,3].

Over the past two decades, Ethiopia has made considerable progress toward malaria control and elimination through the implementation of integrated interventions, including widespread use of insecticide-treated nets (ITNs), indoor residual spraying (IRS), improved diagnostic services, effective case management, and strengthened surveillance systems [3,4]. These efforts have contributed to reductions in malaria incidence and mortality; however, persistent transmission continues to challenge elimination efforts in several parts of the country [2,5].

While malaria control programs have traditionally focused on the detection and treatment of symptomatic infections, increasing evidence highlights the epidemiological importance of asymptomatic malaria infection (AMI) [6–8]. Individuals with AMI harbor malaria parasites without exhibiting clinical symptoms and often remain undetected by routine surveillance systems [6,9]. Consequently, they may serve as reservoirs of infection that sustain ongoing transmission within communities. These silent infections are particularly important in low- and moderate-transmission settings, where undetected parasite carriage can undermine malaria control strategies and hinder progress toward elimination [7,8,10].

School-aged children represent an important but frequently overlooked population in malaria epidemiology [8]. Repeated exposure to malaria parasites can result in the development of partial immunity, allowing infections to persist without symptoms while maintaining low-density parasitemia [11]. Consequently, school-aged children may contribute disproportionately to ongoing transmission [8,12]. Beyond their epidemiological significance, AMI in this age group has been associated with adverse health and educational outcomes, including anemia, impaired cognitive performance, reduced school achievement, and diminished overall well-being [13,14]. Despite these concerns, malaria interventions in Ethiopia have primarily targeted children under five years of age and pregnant women, with limited emphasis on school-aged populations [3].

Several studies conducted in Ethiopia have reported the prevalence of AMI among school-aged children; however, findings vary considerably across regions, diagnostic methods, and years of actual data collection [8,15–17]. Such variability reflects the heterogeneous nature of malaria transmission and complicates efforts to determine the overall burden of infection [11,18,19]. To date, no systematic review and meta-analysis (SRMA) has comprehensively synthesized the available evidence on the prevalence of AMI among school-aged children in Ethiopia. The absence of consolidated evidence limits the ability of policymakers and malaria control programs to design targeted interventions addressing this potential reservoir of infection. Therefore, this study aims to estimate the pooled national prevalence of AMI, the regional distribution of AMI, *Plasmodium* species distribution, and the associated risk factors of AMI among school-aged children in Ethiopia

### Outcomes and prioritization

The primary outcome of this SRMA is the pooled prevalence of laboratory-confirmed AMI among school-aged children in Ethiopia. Secondary outcomes will include: (1) the distribution of *Plasmodium* species among laboratory-confirmed AMI cases, (2) evidence of reported factors associated with AMI among school-aged children, (3) variation in prevalence estimates according to diagnostic method (including polymerase chain reaction (PCR), rapid diagnostic tests (RDTs), and microscopy), study setting (school-based versus community-based), year of publication and years of actual data collection, and transmission season during data collection, which will be classified according to the data-collection period and the established seasonal pattern of malaria transmission in Ethiopia. The major transmission season will be defined as September-December, following the main rainy season, and the minor transmission season as April-May, following the secondary rainy season. Studies conducted outside these periods will be classified as low transmission, while those spanning two or more periods will be classified as mixed transmission [20]. Where available, the season reported by the original study will be retained; otherwise, classification will be based on the reported data-collection months.

When a study reports prevalence estimates based on multiple diagnostic methods for the same study population, a predefined diagnostic-method hierarchy will be applied when selecting the primary prevalence estimate for the overall meta-analysis. PCR-based estimates will be prioritized, followed by RDT and microscopy. This hierarchy will be applied consistently across studies to avoid double-counting participants and to ensure comparability of the primary analysis. However, prevalence estimates obtained using the different diagnostic methods will also be extracted and recorded separately for secondary analyses. Diagnostic method will be considered a potential source of between-study heterogeneity and will be evaluated using subgroup analysis. Sensitivity analyses will also be conducted, where appropriate, to assess the robustness of the pooled prevalence estimates to the choice of diagnostic method.

All outcomes will be considered important for review; however, primary emphasis will be placed on the pooled prevalence estimate, as it represents the central measure of disease burden. Secondary outcomes will be used to complement the primary findings and to provide a more comprehensive understanding of the epidemiology and determinants of AMI in the target population.

### Potential implications for research and policy

The findings of this SRMA are expected to have important implications for both research and public health policy. By synthesizing evidence on the burden of AMI among school-aged children in Ethiopia, the study will provide an updated evidence base to inform malaria control and elimination strategies. From a policy perspective, the results may support the reorientation of malaria prevention programs to give greater attention to school-aged children, who may serve as a significant reservoir for ongoing transmission. The evidence generated could inform the design and implementation of targeted interventions such as school-based malaria screening, health education programs, and strengthened vector control measures in endemic areas [12]. In addition, identifying differences in prevalence by region and diagnostic method may help guide more context-specific and resource-efficient public health strategies. From a research perspective, the review is expected to highlight key evidence gaps, including limited geographic coverage, inconsistencies in diagnostic approaches, and insufficient reporting of age-disaggregated data. These gaps may guide future primary studies, particularly longitudinal and intervention-based research focusing on AMI dynamics in school-aged children. The study may also encourage improved standardization in reporting and diagnostic methodologies to enhance comparability across studies in Ethiopia and similar settings.

## Materials and methods

### Protocol registration and amendments

This protocol was developed in accordance with the Preferred Reporting Items for Systematic Review and Meta-Analysis Protocols (PRISMA-P) guideline (S1 File). The completed systematic review will be reported in accordance with the Preferred Reporting Items for Systematic Reviews and Meta-Analyses (PRISMA) 2020 statement [22,23]. The protocol for this systematic review and meta-analysis was registered in the International Prospective Register of Systematic Reviews (PROSPERO) under registration number CRD420261408867 [24].

Any important amendments to this protocol will be documented with a clear description of the change, the rationale for the modification, and the date of revision. Amendments that may affect the review methodology, including changes to eligibility criteria, search strategy, outcome definitions, data extraction procedures, or statistical analysis plans, will be recorded and updated in the PROSPERO registration record where applicable. All protocol modifications will be reported transparently in the final systematic review publication to ensure methodological transparency and reproducibility. If the review is terminated before completion, the reasons for discontinuation will be documented and reported appropriately.

### Information sources and search strategy

A comprehensive literature search will be conducted to identify studies reporting the prevalence, Plasmodium species distribution, and associated factors of AMI among school-aged children in Ethiopia. The literature search will include all eligible studies published from database inception through June 2026. The electronic search will be conducted from June 10 to June 30, 2026. The search will be updated immediately before final manuscript submission to identify any newly published or relevant studies. The electronic databases to be searched will include PubMed, Scopus, CINAHL, and ScienceDirect. To maximize the identification of potentially eligible studies and reduce the risk of missing relevant evidence, additional searches will be conducted in Google Scholar, African Journals Online (AJOL), Semantic Scholar, and Academia.edu. Grey literature will also be sought from institutional repositories and other relevant academic sources. These records will be considered only when sufficient methodological and outcome information is available to assess eligibility and risk of bias. All identified records will undergo the same predefined screening and eligibility assessment. When a grey-literature record has a corresponding peer-reviewed publication, the peer-reviewed version will be prioritized for data extraction.

The search strategy (S2 File) will employ a combination of controlled vocabulary terms (Medical Subject Headings [MeSH] and database-specific subject headings) and free-text keywords related to malaria, asymptomatic infection, parasite carriage, school-aged children, epidemiological outcomes, and Ethiopia. The main search concepts will include: (1) malaria and Plasmodium species (“Malaria,” malaria, Plasmodium, “Plasmodium falciparum,” “Plasmodium vivax”); (2) asymptomatic or subclinical infection (asymptomatic, “asymptomatic infection,” subclinical, “sub-clinical,” submicroscopic, “sub-microscopic,” parasitemia, and “malaria parasite”); (3) children and adolescents (child*, adolescent*, “school child*,” “school-age child*,” “school-age children,” and student*); (4) epidemiological and outcome-related terms (prevalence, burden, epidemiology, frequency, occurrence, risk factor*, predictor*, determinant*, and associated factor*); and (5) Ethiopia. Boolean operators (“AND” and “OR”), phrase searching, truncation, and database-specific search functions will be applied appropriately. The search strategy will be adapted according to the requirements of each electronic database. Additionally, reference lists of all eligible studies and relevant systematic reviews will be manually screened to identify additional potentially eligible studies.

### Records and data management

All records identified through electronic database searches will be imported into Mendeley Reference Manager (Elsevier) for citation management and to remove duplicates, eliminate multiple versions of the same study, and exclude irrelevant records and those that do not meet the predefined study-design and outcome criteria. Following deduplication, the remaining references will be uploaded to Rayyan, a web-based systematic review platform, to facilitate title, abstract, and full-text screening by independent reviewers [21]. Screening decisions, reasons for exclusion at the full-text stage, and reviewer disagreements will be documented and retained within the platform. Data extraction and risk-of-bias assessments will be conducted using standardized, pilot-tested electronic data extraction forms. All extracted data, assessment files, and review documents will be stored in password-protected electronic folders with regular backups. Version control procedures will be implemented to ensure transparency and traceability of any modifications made during the review process. Only members of the review team will have access to the review data throughout the study.

### Article eligibility criteria

Study eligibility for this review will be determined using the CoCoPop (Condition, Context, Population) framework recommended by the Joanna Briggs Institute for prevalence reviews [25,26]. The condition of interest will be AMI, defined as the laboratory-confirmed presence of malaria parasites in the blood of individuals who do not exhibit clinical signs or symptoms of malaria at the time of the investigation. The context will be limited to studies conducted in Ethiopia. The target population will comprise school-aged children aged 5–16 years. Studies will be eligible for inclusion if they are community- or school-based observational studies (cross-sectional or cohort) that report the prevalence, Plasmodium species distribution, and/or risk factors of AMI. Studies focusing exclusively on adult populations, children under five years of age, pregnant women, or individuals seeking care for active, symptomatic clinical malaria cases will be excluded (Table 1).

**Table 1:** Inclusion and exclusion criteria for the SRMA of AMI among school-aged children in Ethiopia.

Category	Inclusion Criteria	Exclusion Criteria
Population	Studies involving schoolchildren aged 5–16 years, or studies with a broader age range from which data specific to children aged 5–16 years can be extracted.	Studies that do not provide separate or extractable data for children aged 5–16 years.
Condition	Studies reporting laboratory-confirmed AMI	Studies focusing exclusively on symptomatic malaria cases or clinical malaria.
Context	Studies conducted in Ethiopia	Studies conducted outside Ethiopia
Study Design	Observational studies (cross-sectional or cohort studies)	Reviews, editorials, commentaries, case reports, case series, qualitative studies, and intervention studies without baseline prevalence data
Outcomes	Studies reporting AMI prevalence and/or Plasmodium species distribution and/or factors associated with AMI among school-aged children.	Studies without extractable outcome data relevant to AMI among school-aged children.
Language	Studies published in English language	Non-English language publications
Data Availability	Full-text studies provide sufficient methodological and outcome information for data extraction and assessment of study quality.	Studies with unavailable full text, insufficient information, or inadequate data for extraction after reasonable attempts to obtain the required information.

To ensure comprehensive coverage of the available evidence, studies conducted in the general population will also be considered eligible if they provide primary, age-disaggregated data for schoolchildren aged 5–16 years separately. Such studies must explicitly report both the total number of school-aged children and the corresponding number of malaria-positive cases within that subgroup. Studies that report only aggregate age measures, such as mean or median age, without subgroup-specific outcome data will be excluded. Moreover, multiple publications from the same study population will be considered when they provide distinct outcomes or subgroup data; overlapping populations will be identified to avoid double-counting participants. For analyses of *Plasmodium* species distribution and risk factors, studies will be included only if outcome data for school-aged children can be extracted independently. When age-stratified prevalence data are available, but species-specific prevalence or risk factor estimates are reported only for the overall study population, the study will be included in the overall prevalence meta-analysis but excluded from the corresponding subgroup analyses. Where necessary, corresponding authors will be contacted to obtain age-specific data. If the requested data cannot be obtained, the study will be excluded from the relevant quantitative synthesis and considered for narrative description.

No restriction will be placed on year of publication. Only studies published in English and available as full-text articles, reports, theses, or dissertations will be considered. This restriction is applied due to resource limitations for translation and because most of the Ethiopian malaria research is published in English. Studies with insufficient methodological information, inaccessible full texts, duplicate publications, suspected data fabrication, or serious ethical concerns will be excluded. When multiple publications report data from the same study population, the most comprehensive and relevant data for the prespecified outcome and population will be retained to avoid double-counting participants and inflating pooled estimates. To ensure methodological consistency, studies will be required to employ recognized malaria diagnostic methods. Furthermore, diagnostic methods must be applied consistently across age groups within individual studies when age-stratified estimates are extracted.

### Study selection and screening

Two reviewers will independently screen titles and abstracts of all retrieved records in Rayyan, blinded to each other’s decisions during initial screening. Records deemed potentially eligible by either reviewer will proceed to full-text assessment. Reviewers will resolve screening disagreements through discussion and consensus. If consensus cannot be reached, a third senior reviewer will make the final decision. Two other reviewers will retrieve the full texts of potentially eligible studies and independently assess them against the predefined inclusion and exclusion criteria. For studies with missing, unclear, or incomplete information, corresponding authors will be contacted via email to obtain additional details or clarification. We will contact authors twice over three weeks. If no response is received within the specified period, the study will be analyzed using the available information, where appropriate, or the relevant data point will be excluded from the analysis. The study selection process will be documented and reported using a PRISMA 2020 flow diagram (Fig 1). Reasons for excluding studies at the full-text review stage will be recorded systematically and presented in a supplementary table. Studies reporting the prevalence of AMI among school-aged children with sufficient data to estimate prevalence will be included in the quantitative synthesis. Meta-analysis will be conducted when at least two comparable studies are available.

**Fig 1:**
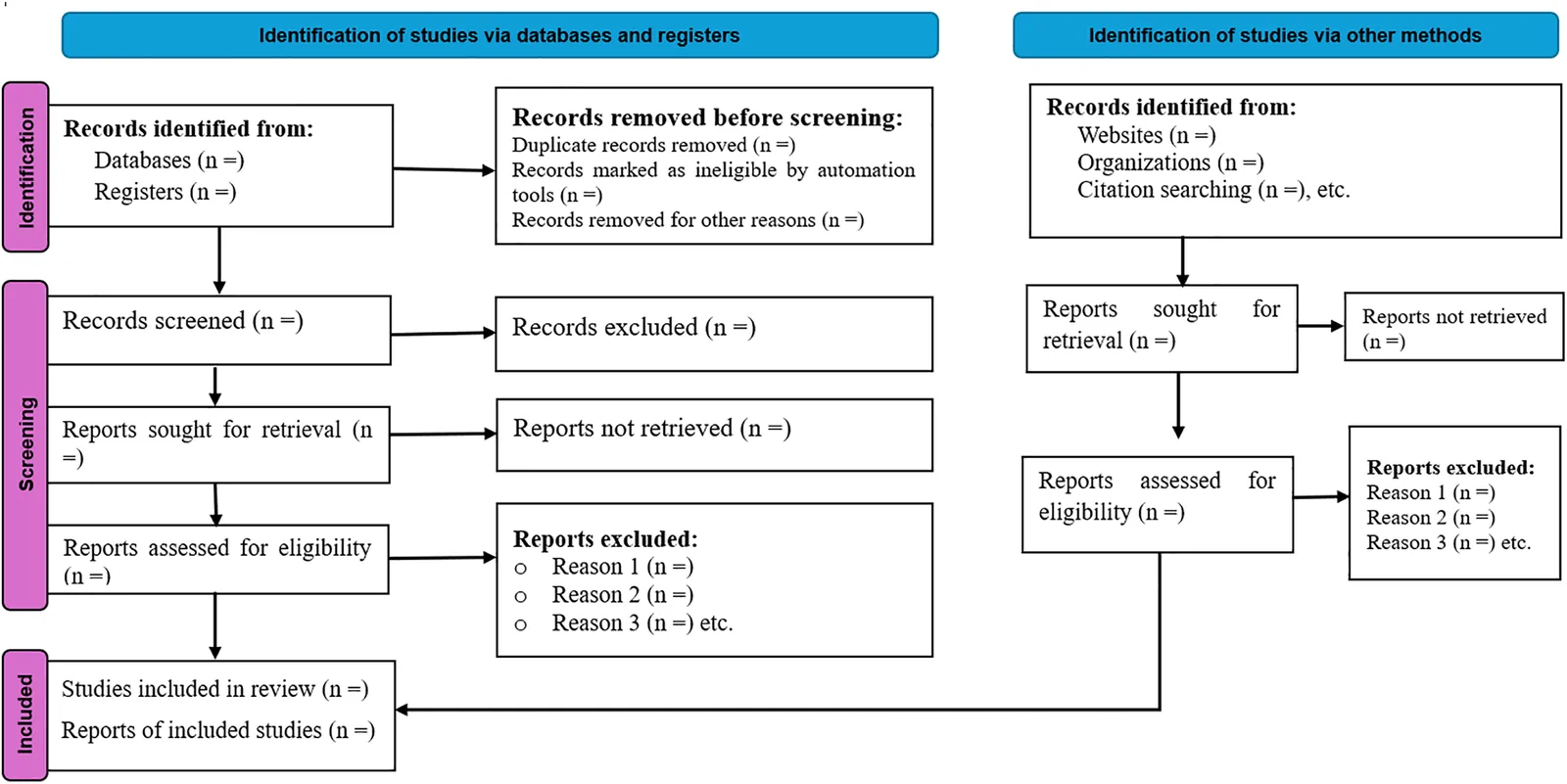
PRISMA flow diagram for study identification, screening, eligibility assessment, and final inclusion in the SRMA of AMI among school-aged children.

If meta-analysis is not appropriate due to substantial clinical, methodological, or statistical heterogeneity, insufficient data, or an inadequate number of comparable studies, the findings will be synthesized narratively. The narrative synthesis will summarize study characteristics, methodological quality, diagnostic methods, geographic distribution, and reported prevalence estimates of asymptomatic malaria infection among school-aged children. Results will be presented in structured tables and descriptive text, with studies grouped according to relevant characteristics such as region, transmission setting, diagnostic method, and years of actual data collection to facilitate comparison and interpretation of findings.

### Data extraction

Data from all eligible studies will be extracted using a standardized and pre-piloted data extraction form (S3 File). The extraction process will be conducted independently by two reviewers to ensure accuracy, consistency, and completeness of the collected information. Any discrepancies between the reviewers will be resolved through discussion and consensus, and when necessary, a third reviewer will be consulted to reach a final decision. As outlined in Supplementary S3 File, the extracted data will include study-level characteristics such as the first author, year of publication, year of actual data collection, and the geographic area where the study was conducted. Additional methodological details will include the study design, study setting (community-based or school-based), sampling method, the characteristics of the study population, response rate, and the total sample size. Outcome-related data will also be extracted, including the number of participants tested for malaria, the number of AMI-positive cases, the diagnostic methods used, malaria species, and the reported prevalence of AMI. Where available, age-stratified data for school-aged children will be extracted separately for inclusion in subgroup analyses; similarly, AMI-associated factor-related data like age, sex, transmission season during data collection, altitude/ecological zone, urban/rural, definition of asymptomatic infection, antimalarial treatment/exclusion criteria, ITN use where reported, IRS exposure where reported, socioeconomic variables, and adjusted risk estimates will be extracted.

### Data synthesis and statistical analysis

All extracted data will be checked for completeness, consistency, and accuracy before being entered into a structured database for analysis. Descriptive statistics will summarize the characteristics of included studies in tabular form. The pooled prevalence of AMI will be estimated using a generalized linear mixed-effects model (GLMM) with a binomial distribution and logit link, implemented in R (version 4.5.2) using the meta and/or metafor package. The specific versions of the meta and/or metafor packages used will be recorded and reported to ensure reproducibility. The model will account for within-study sampling variability and between-study heterogeneity through a study-level random effect. Pooled estimates will be back-transformed from the logit scale to the proportion scale and presented with corresponding 95% confidence intervals. Forest plots will be generated to display individual study prevalence estimates and the overall pooled prevalence.

For risk-factor analyses, adjusted effect estimates (adjusted odds ratios [AORs]) and their corresponding 95% confidence intervals will be extracted whenever available. Where at least two studies report AORs for the same risk factor with sufficiently comparable exposure and outcome definitions, reference categories, and adjustment approaches, the estimates will be log-transformed and quantitatively synthesized using a random-effects model. When quantitative synthesis is not appropriate because of an insufficient number of studies, inconsistent definitions, or substantial methodological heterogeneity, findings will be summarized narratively.

### Assessment of heterogeneity and sensitivity analysis

Statistical heterogeneity among studies will be assessed using Cochran’s Q test, with a p-value < 0.05 indicating significant heterogeneity. Also, the magnitude of heterogeneity will be quantified using the I^2^ statistic, with values of 25%, 50%, and 75% interpreted as low, moderate, and high heterogeneity, respectively [27]. Between-study heterogeneity will be incorporated into the generalized linear mixed-effects model through a study-level random effect. Forest plots will be used to visually examine variability in prevalence estimates across studies, while Galbraith plots will be constructed to identify potential outliers and further explore sources of heterogeneity.

When substantial heterogeneity is observed and sufficient data are available, predefined subgroup analyses will be conducted. These will be based on diagnostic method, study setting (school-based versus community-based), study area/region, transmission season during data collection, study design, and years of data collection. For studies reporting prevalence estimates using multiple diagnostic methods within the same study population, data will be extracted and analyzed separately within the corresponding diagnostic subgroups to avoid double-counting participants and unit-of-analysis errors. For the primary pooled prevalence analysis, only one prevalence estimate will be included from each study population. When multiple diagnostic methods are reported for the same participants, the estimate derived from the most sensitive diagnostic method will be selected for the overall analysis, while estimates from all reported diagnostic methods will be retained for diagnostic-specific subgroup analyses. The robustness of the pooled prevalence estimates will be assessed through sensitivity analyses. A leave-one-out analysis will be conducted by sequentially excluding individual studies and recalculating the pooled estimate. Additional sensitivity analyses will be performed by excluding studies judged to be at high risk of bias to evaluate their influence on the overall findings [28].

### Meta-regression and temporal trends

Random-effects meta-regression will also be conducted to explore potential sources of between-study heterogeneity. Approximately 10 or more studies per covariate will be considered when selecting moderators for meta-regression to reduce the risk of overfitting. Prespecified study-level moderators will include diagnostic method, study setting, study area, transmission season, study design, and year of actual data collection. Potential temporal trends in AMI prevalence will be explored using the year of actual data collection as a continuous moderator. When the number of studies is insufficient for meta-regression, temporal patterns will be explored descriptively by year of data collection [28].

### Assessment of small-study effects and publication bias

Small-study effects and potential publication bias will be evaluated visually using funnel plots, while statistical evaluation will be conducted using Egger’s regression test and Begg’s rank correlation test when an adequate number of studies are available [29]. A p-value < 0.05 will be considered indicative of statistically significant asymmetry. However, funnel plot asymmetry will be interpreted cautiously because it may result from factors other than publication bias. Additionally, Doi plots with the Luis Furuya-Kanamori (LFK) index will be used to quantify asymmetry [30]. These analyses will be conducted using the metasens package in R, which implements both the Doi plot and LFK index. An LFK index between −1 and +1 will be interpreted as no asymmetry, values between ±1 and ±2 as minor asymmetry, and values beyond ±2 as major asymmetry. Where evidence of asymmetry is observed, sensitivity analysis using the trim-and-fill method will be performed to explore the potential impact of missing studies on the pooled prevalence estimate. The adjusted estimates will be compared with the primary analysis to evaluate the robustness and stability of the findings.

### Assessment of certainty of evidence

The certainty of evidence for the main outcomes will be assessed using the Grading of Recommendations, Assessment, Development and Evaluation (GRADE) approach adapted for prevalence studies [31,32]. The overall certainty of evidence will be rated as high, moderate, low, or very low based on the GRADE domains of risk of bias, inconsistency, indirectness, imprecision, and publication bias. For prevalence outcomes, the certainty of evidence will be evaluated according to recommendations for rating evidence from observational studies [33] while adhering to current requirements for the application and reporting of GRADE [34]. Summary of Findings tables will be prepared to present the overall certainty ratings and the rationale for any decisions to downgrade or upgrade the certainty of evidence.

### Quality assessment and risk of bias analysis

The methodological quality and risk of bias of all included studies will be assessed independently by two reviewers using the JBI Critical Appraisal Checklist for Studies Reporting Prevalence Data (S4 File). This tool evaluates nine methodological domains, including the appropriateness of the sampling frame, participant recruitment procedures, adequacy of sample size, detailed description of study subjects and settings, coverage of the identified sample in the analysis, validity and reliability of methods used to detect AMI, consistency of measurement across participants, appropriateness of statistical analyses, and adequacy of the response rate [26].

Each checklist item will be rated as “Yes,” “No,” “Unclear,” or “Not Applicable,” in accordance with JBI guidance. Risk of bias will be assessed at the individual domain level [35]. Attention will be given to the representativeness of the study population, sampling procedures, completeness of outcome data, and the diagnostic methods used to identify AMI. Disagreements between the two reviewers will be resolved through discussion and consensus. Inter-reviewer agreement before consensus will be assessed using Cohen’s kappa statistic (κ). A value of κ > 0.60 will be considered indicative of acceptable agreement [36]. If consensus cannot be achieved, a third reviewer will be consulted. The results of the methodological quality and risk-of-bias assessment will be presented in tabular form and incorporated into the interpretation of the review findings.

### Ethical considerations and declarations

This SRMA will not require ethical approval from a research ethics review board, as it will rely exclusively on data extracted from previously published studies and will not involve direct contact with human participants or the collection of individual-level primary data. Also, contacting primary authors for aggregate, unpublished subgroup data does not require new ethical clearance. Despite this exemption, the review will be conducted in accordance with the ethical principles outlined by the Committee on Publication Ethics (COPE) [37]. These guidelines will be followed to ensure integrity, transparency, and responsible reporting throughout the review process, including accurate citation of sources and appropriate handling of published data.

### Study status and timeline

This systematic review and meta-analysis protocol was developed before the initiation of the study selection. The review team has completed the development of the research question, eligibility criteria, methodological framework, and preliminary search strategy. The planned timeline for the review activities is as follows: protocol development and registration was conducted from March 4 to June 9, 2026; the literature search will be performed from June 10 to June 30, 2026; title and abstract screening will be conducted from July 1–20, 2026; full-text assessment will be completed from July 21 to August 5, 2026; data extraction and risk-of-bias assessment will be undertaken from August 6 to August 20, 2026; data synthesis and statistical analysis will be performed from August 21 to September 10, 2026; and manuscript preparation and submission are planned for September 11 to September 20, 2026.

## Discussion

Ethiopia has adopted an ambitious malaria elimination agenda and has achieved substantial reductions in malaria morbidity and mortality through the scale-up of vector control interventions, improved diagnostic capacity, artemisinin-based combination therapy, and strengthened surveillance systems [2–4]. Nevertheless, progress toward elimination remains uneven, as persistent transmission continues in several endemic regions. National strategy now prioritizes identifying the hidden reservoirs of infection that evade routine, symptom-driven surveillance [3,6]. In this context, AMI is a critical, yet under-characterized, threat to these elimination goals [6,10,19].

A challenge in synthesizing evidence on AMI may be the methodological heterogeneity across studies, particularly differences in diagnostic methods (conventional microscopy and rapid RDTs to highly sensitive molecular techniques) and transmission settings [6,10,19,38]. Variations in diagnostic sensitivity may contribute substantially to differences in reported prevalence estimates. To address this, our protocol incorporates a comprehensive data extraction strategy that will allow for subgroup analyses, meta-regression, and sensitivity analyses conducted to explore potential sources of heterogeneity. By stratifying findings, this review aims to clarify whether observed variations in prevalence reflect genuine epidemiological differences or are artifacts of diagnostic sensitivity. Despite limitations related to diagnostic variability, study heterogeneity, restricting the review to English-language articles, and possible publication bias, this review will provide the first comprehensive synthesis of AMI among Ethiopian school-aged children. The findings are expected to clarify the epidemiological role of this population as a malaria reservoir and provide evidence to support targeted surveillance and school-based malaria interventions within Ethiopia’s elimination strategy [8,11].

## Conclusion

This SRMA will provide a comprehensive synthesis of the burden and epidemiological characteristics of AMI among school-aged children in Ethiopia. By consolidating evidence across regions and study settings, it will generate more precise estimates and identify key patterns in transmission dynamics. The findings are expected to inform national malaria elimination strategies by highlighting the epidemiological importance of school-aged children as a potential reservoir of infection and by identifying gaps in surveillance and intervention coverage

## Supporting information

S1 File PRISMA-P-checklist. (DOCX)

S2 File Full search strategy for all databases. (DOCX)

S3 File Data extraction tool for systematic review and meta-analysis. (DOCX)

S4 File Risk of bias assessment tool. (DOCX)

## Abbreviations

AMI: Asymptomatic Malaria Infection
JBI: Joanna Briggs Institute
MeSH: Medical Subject Headings
PCR: Polymerase Chain Reaction
PRISMA: Preferred Reporting Items for Systematic Reviews and Meta-Analyses
RDT: Rapid Diagnostic Test
SRMA: systematic review and meta-analysis
