# Supplementary material for: Burden of asymptomatic malaria among school-aged children: Protocol for a systematic review and meta-analysis
Source: PLoS One. 2026 Sep 30;21(9):e0359714. doi: 10.1371/journal.pone.0359714 (PMC13626321; doi:10.1371/journal.pone.0359714)
Supplement: Supplementary file 2 [file pone.0359714.s002.docx]

**Supplementary Table S1.** Detailed Search Strategies Used for Electronic Databases

**Database:** PubMed/MEDLINE(NLM)

**Search String:** (("Malaria"[Mesh] OR malaria OR plasmodium OR "Plasmodium falciparum" OR "Plasmodium vivax") AND (asymptomatic OR "asymptomatic infection" OR subclinical OR "sub-clinical" OR submicroscopic OR "sub-microscopic" OR parasitemia OR "malaria parasite") AND (child* OR adolescent* OR "school child*" OR "school-age child*" OR "school-age children" OR student*) AND (prevalence OR burden OR epidemiology OR occurrence OR predictor* OR determinant* OR "associated factor*" OR "risk factor*" ) AND (Ethiopia[Mesh] OR Ethiopia) )

**Data Base: Scopus (Elsevier)**

**Search String:** TITLE-ABS-KEY ( ( malaria OR plasmodium OR "Plasmodium falciparum" OR "Plasmodium vivax" ) AND ( asymptomatic OR "asymptomatic infection" OR subclinical OR "sub-clinical" OR submicroscopic OR "sub-microscopic" OR parasitemia OR "malaria parasite" ) AND ( child* OR adolescent* OR "school child*" OR "school-age child*" OR "school-age children" OR student* ) AND ( prevalence OR burden OR epidemiology OR frequency OR occurrence OR determinant* OR predictor* OR "associated factor*" OR "risk factor*" ) AND (Ethiopia OR Ethiopian*))

**Database:** CINAHL (EBSCOhost)

**Search String:** ((MH "Malaria+") OR malaria OR plasmodium OR "Plasmodium falciparum" OR "Plasmodium vivax") AND (asymptomatic OR "asymptomatic infection" OR subclinical OR "sub-clinical" OR submicroscopic OR "sub-microscopic" OR parasitemia OR "malaria parasite") AND (child* OR adolescent* OR "school child*" OR "school-age child*" OR "school-age children" OR student*) AND (prevalence OR burden OR epidemiology OR frequency OR occurrence OR determinant* OR predictor* OR associated factor OR "risk factor*") AND (Ethiopia OR Ethiopian*)

**Database:** **ScienceDirect (Elsevier)**

**Search String:** (malaria OR “plasmodium) AND (asymptomatic OR subclinical OR submicroscopic) AND (schoolchildren OR “school-age children”) AND Ethiopia

Google Scholar

**Search String:** intitle:asymptomatic malaria Ethiopia (children OR adolescent OR school children OR school age children)
