# Supplementary material for: Burden of asymptomatic malaria among school-aged children: Protocol for a systematic review and meta-analysis
Source: PLoS One. 2026 Sep 30;21(9):e0359714. doi: 10.1371/journal.pone.0359714 (PMC13626321; doi:10.1371/journal.pone.0359714)
Supplement: Supplementary file 3 [file pone.0359714.s003.docx]

Supplementary File 3: Data Extraction Tool for Systematic Review and Meta-analysis

| No | Required item |
| --- | --- |
| 1 | Full title of the article: ________________________________________________________________________ |
| 2 | Identification number assigned during screening: __________________________ |
| 3 | Year the article was published: ________________________________________ |
| 4 | Name of the journal or publication source: _______________________________ |
| 5 | Name of the first author: _____________________________________________ |
| 6 | Corresponding author email address: ___________________________________ |
| 7 | Was the author contacted? (Yes/No). If yes, when was the author contacted, and what information was obtained? |
| 8 | Year(s) of data collection: ____________________________________________ |
| 9 | Where was the study conducted (region and specific study site/town)? |
| 10 | When did data collection start and end? |
| 11 | When was the study conducted (season/month of data collection)? |
| 12 | How was the study area classified? (Urban / Rural / Mixed / Other: ____________________________) |
| 13 | Altitude category of the study area (if reported): _______________________________________________ |
| 14 | Was the study design clearly described? (Yes/No). If yes, specify: ______________________________________________________________________________________ |
| 15 | Where was the study conducted? (Community / School / Hospital / Other: __________________________________) |
| 16 | Was the sampling method clearly described? (Yes/No). If yes, specify: _____________________________________ |
| 17 | Was the sample size calculated? (Yes/No). If yes, what was the calculated sample size? _____ Was the calculated sample size adequate? (Yes/No)" |
| 18 | Was sufficient information provided about the sampling frame? (Yes/No) |
| 19 | Who was the target population of the study? |
| 20 | Participant age range: _____________________________________________________________________ |
| 21 | Participant age (mean ± SD or median, if reported): _____________________________________________ |
| 22 | Sex distribution of participants: Male (n) __________________; Female (n) __________________________ |
| 23 | Study inclusion criteria: ____________________________________________________________________ |
| 24 | Study exclusion criteria: ____________________________________________________________________ |
| 25 | Did the study provide a clear definition of asymptomatic malaria?" (Yes/No) |
| 26 | How many diagnostic methods were used? |
| 27 | Number of positive cases detected by the most sensitive diagnostic method |
| 28 | Was microscopy performed? (Yes/No). If yes, number of microscopy-positive cases (n): _____________________________ |
| 29 | Number of microscopy-positive cases by species: Pf (n) ___; Pv (n) ___; Mixed infection (n) ___; Other species (n) ___. |
| 30 | Was RDT performed? (Yes/No). If yes, number of RDT-positive cases (n): ____________________________________________ |
| 31 | Number of RDT-positive cases by species: Pf (n) ___; Pv (n) ___; Mixed infection (n) ___; Other species (n) ___. |
| 32 | Was PCR performed? (Yes/No). If yes, number of PCR-positive cases (n): ____________________________ |
| 33 | Number of RDT-positive cases by species: Pf (n) ___; Pv (n) ___; Mixed infection (n) ___; Other species (n) ___. |
| 34 | Did the study use a standard data collection tool? (Yes/No) |
| 35 | Was laboratory quality control performed and reported? (Yes/No). If yes, specify: _____________________ |
| 36 | Name of the predictor variable associated with the outcome" |
| 37 | Were exposure variables clearly defined and described?" (Yes/No) |
| 38 | Number of exposed participants with outcome / total exposed participants (n/N) |
| 39 | Number of participants with the outcome among the unexposed group / total unexposed participants (n/N) |
| 40 | Was the adjusted odds ratio (AOR) reported? (Yes/No). If yes, reported AOR: ______________ |
| 41 | Was the 95% confidence interval (CI) reported? (Yes/No). If yes, report the lower and upper CI limits: ____________ |
| 42 | Which statistical approach was used to analyze the data? |
| 43 | Were confounders controlled? (Yes/No) |
| 44 | Which category was used as the reference group? |
| 45 | Was the response rate reported? (Yes/No). If yes, response rate (%): ___________________________ |
| 46 | Were missing data addressed in the analysis? (Yes/No). If yes, specify the method used: _____________________ |
| 47 | Was ethical approval obtained? (Yes/No)" |
| 48 | Were study limitations and potential sources of bias described? (Yes/No). If yes, specify: ___________________ |
| 49 | Did the authors disclose any potential conflicts of interest? (Yes/No). If yes, specify: _____________________________________ |
