# Supplementary material for: Burden of asymptomatic malaria among school-aged children: Protocol for a systematic review and meta-analysis
Source: PLoS One. 2026 Sep 30;21(9):e0359714. doi: 10.1371/journal.pone.0359714 (PMC13626321; doi:10.1371/journal.pone.0359714)
Supplement: Supplementary file 4 [file pone.0359714.s004.docx]

Supplementary File 4: Joanna Briggs Institute (JBI) Critical Appraisal Checklist for Prevalence Studies

| No | JBI Item | Yes | No | Unclear | NA |
| --- | --- | --- | --- | --- | --- |
| 1 | Was the sample frame appropriate to address the target population? |  |  |  |  |
| 2 | Were study participants sampled in an appropriate way? |  |  |  |  |
| 3 | Was the sample size adequate? |  |  |  |  |
| 4 | Were the study subjects and setting described in detail? |  |  |  |  |
| 5 | Was the data analysis conducted with sufficient coverage of the identified sample? |  |  |  |  |
| 6 | Were valid methods used for the identification of the condition? |  |  |  |  |
| 7 | Was the condition measured in a standard, reliable way for all participants? |  |  |  |  |
| 8 | Was there an appropriate statistical analysis? |  |  |  |  |
| 9 | Was the response rate adequate, and if not, was the low response rate managed appropriately? |  |  |  |  |
